# Rapid and Liquid-Based Selection of Genetic Switches Using Nucleoside Kinase Fused with Aminoglycoside Phosphotransferase

**DOI:** 10.1371/journal.pone.0120243

**Published:** 2015-03-19

**Authors:** Masahiro Tominaga, Kohei Ike, Shigeko Kawai-Noma, Kyoichi Saito, Daisuke Umeno

**Affiliations:** 1 Department of Applied Chemistry and Biotechnology, Faculty of Engineering, Chiba University, 1–33 Yayoi-Cyo, Inage-ku, Chiba, Japan; 2 Precursory Research for Embryonic Science and Technology (PRESTO), Japan Science and Technology Agency (JST), 4–1–8 Honcho, Kawaguchi, Saitama, Japan; The Scripps Research Institute, UNITED STATES

## Abstract

The evolutionary design of genetic switches and circuits requires iterative rounds of positive (ON-) and negative (OFF-) selection. We previously reported a rapid OFF selection system based on the kinase activity of herpes simplex virus thymidine kinase (hsvTK) on the artificial mutator nucleoside dP. By fusing hsvTK with the kanamycin resistance marker aminoglycoside-(3’)-phosphotransferase (APH), we established a novel selector system for genetic switches. Due to the bactericidal nature of kanamycin and nucleoside-based lethal mutagenesis, both positive and negative selection could be completed within several hours. Using this new selector system, we isolated a series of homoserine lactone-inducible genetic switches with different expression efficiencies from libraries of the *Vibrio fischeri lux* promoter in two days, using only liquid handling.

## Introduction

Genetic switches and their assemblies (regulatory circuits) consist of multiple interacting component functions. Such multi-body functions are highly context-dependent and resistant to rational design. Evolutionary design, *i*.*e*., library generation followed by functional selection, has therefore been widely used to create or improve genetic switches [1,2].

Irrespective of component number and type, genetic switches and circuits are ultimately a triggering device between ON/OFF states. Thus, selection of their function can easily be accomplished by coupling the device output to the expression of two independent genes: a rescuer (ON-selector) and a killer (OFF-selector) [3,4].

To truly facilitate the fast-track construction of genetic switches and circuits, all selection steps should be completed with liquid handling to permit the parallel operation of multiple projects in a multi-well format. In addition, the selection process should be as short as possible, particularly for the construction of complex genetic systems, such as logic gates with multiple inputs possessing 2^n^ independent states (n = the number of inputs) for which evolutionary circuit designers must conduct either ON- or OFF-selection for each of these states. It is also desirable that both ON/OFF- selection reside in a single gene: single-gene dual selectors are also crucial for expanding the application scope of the evolutionary design of genetic switches to *cis*-regulating switches (*e*.*g*., riboswitches and riboregulators) and the *de novo* construction of eukaryotic genetic switches.

Some of the above requirements have been addressed. For instance, single genes that serve as both an ON- and OFF-selectors have been developed to eliminate the need for two independent selector genes [5–9]. TetA, a tetracycline/H^+^ antiporter, functions as an ON-selector upon the addition of tetracycline but functions as an OFF-selector in the presence of toxic metal salts such as NiCl_2_ [5,6]. Gallivan and colleagues devised an alternative type of dual selector system in which CheZ, the core part of a chemotaxis decision-making device, served as both an OFF- and ON-selector [7]. However, these systems exhibit several weaknesses. CheZ require solid media and overnight growth for selection. TetA selection can be conducted in liquid media, but it requires overnight growth in both ON- and OFF-selection.

We recently reported another single-gene dual selector system in which herpes simplex virus thymidine kinase (hsvTK), which has been used as a marker in gene therapy [10], was adapted for the OFF-selection of a genetic circuit [9]. In this system, the mutagenic nucleoside dP [11] was used instead of chain-terminating nucleosides such as ganciclovir and acyclovir [10] to enable OFF (negative)-selection to conditionally kill cells harboring hsvTK with unprecedented speed, efficiency, and selectivity in liquid media [9]. However, the ON-selection of this hsvTK system requires overnight shaking incubation. Another weak point of this system is the requirement for a specific mutant allele (*tdk*-) for ON-selection.

An alternative strategy is to fuse a positive selector gene with a negative selector gene via appropriate peptide linkers to integrate both functions in a single reading frame. Such dual selectors have been developed for conducting positive/negative selection in eukaryotic cells [12,13] to enrich clones with modified chromosome as designed. We hypothesized that they could be also invaluable for seamlessly and rapidly conducting ON-selection (positive selection) and OFF-selection (negative selection) in series. Here, we tested the fusion of hsvTK with conventional positive selection markers, aminoglycoside-(3’)-phosphotransferase (APH) and chloramphenicol acetyltransferase (CAT), and found that the former satisfies the above three requirements. The hsvTK::APH fusion selector was successfully applied to the rapid isolation of a set of variant *Vibrio fischeri* (*V*. *fischeri*) *lux* promoters with different expression efficiencies.

## Materials and Methods

### Materials

3-Oxo-hexanoyl-homoserine lactone (3OC6-HSL) was purchased from Sigma-Aldrich (St. Louis, MO, USA). Stock solutions (1–10 mM) were prepared by dissolving appropriate amounts of 3OC6-HSL in ethyl acetate (Nacalai Tesque, Kyoto, JP) acidified with glacial acetic acid (0.01% (v/v); Nacalai Tesque, Kyoto, JP) and stored at -20°C. 6-(β-D-2-Deoxyribofuranosyl)-3,4-dihydro-8*H*-pyrimido[4,5-c][1,2]oxazin-7-one (dP) was purchased from Berry and Associates (Dexter, MI, USA). Stock solutions (1–1,000 μM) were prepared by dissolving appropriate amounts of dP in DMSO as 1,000 × stock (stored at 4°C). The oligonucleotides used in this work were all synthesized by FASMAC Co., Ltd (Kanagawa, JP). All other chemicals and media were of the highest available grade. Antibiotics were added to the growth medium as required at the following concentrations: 50 μg/mL carbenicillin (Carb; Sigma-Aldrich, St. Louis, MO, USA), 30 μg/mL chloramphenicol (Cm; Nacalai Tesque, Kyoto, JP), and 50 μg/mL kanamycin (Km; Sigma-Aldrich, St. Louis, MO, USA).

### Strains and Plasmids

*Escherichia coli* (*E*. *coli*) strain K-12 MG1655 was used throughout this study, although *E*. *coli* strains XL10-Gold-Kan^r^ (Stratagene, La Jolla, CA, USA) and DH5α (SciTrove, Tokyo, JP) were used for plasmid construction. All plasmids used in this study are shown in S1 Table. Plasmids expressing hsvTK::APH and hsvTK::CAT were constructed as follows. The reading frames of the positive markers *aph* and *cat*, excluding the start codons, were amplified by PCR and fused to the C-terminus of PCR-amplified *hsvtk* without a stop codon in a pJ204 vector (DNA2.0, Menlo Park, CA) with T5/*lacO* promoter and RBS sequence. The *lux* promoter (*plux*) and reading frame of super folder green fluorescent protein (*sfgfp*) were sub-cloned into a pACmod vector [14]. PCR-amplified *hsvtk*::*aph* was sub-cloned in the resulting plasmid, yielding pAC-*plux*-*hsvtk*::*aph*-*sfgfp*. The LuxR expression plasmid was constructed by sub-cloning the PCR-amplified reading frame of LuxR into a pTrcHis2-TOPO vector (Invitrogen, Carlsbad, CA, USA).

### OFF-selection (dP-Selection)

**Fig 1:**
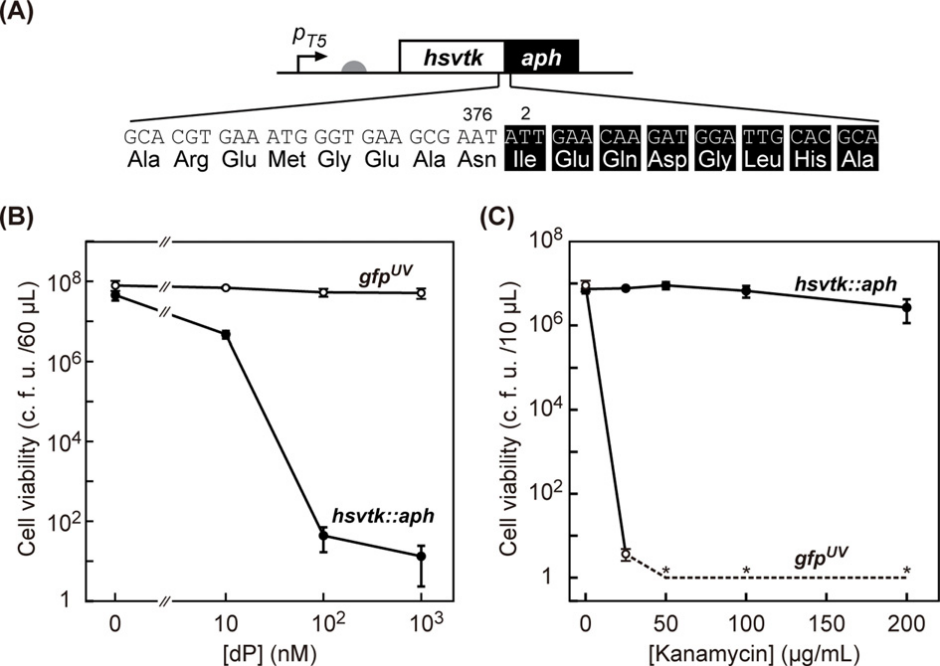
*hsvtk*::*aph* fusion gene as an ON-/OFF-selector. (A) The construct for the expression of hsvTK::APH. The reading frames of hsvTK (excluding stop codon, 376 aa) and APH (excluding the start codon, 263 aa) were fused without a linker, resulting in *hsvtk*::*aph*. The fusion gene was placed under the T5 promoter (*p*_*T5*_) with the translation initiation site (rbs score [25], 13843). (B) OFF-selection using hsvTK::APH selector. *E*. *coli* MG1655 harboring either a plasmid expressing hsvTK::APH or a plasmid expressing GFP^UV^ (negative control) were incubated with dP (0–1,000 nM), and the number of viable (colony forming) cells was measured after 3 h incubation. (C) ON-selection. *E*. *coli* MG1655 harboring either of the plasmids were treated with Km (0–200 μg/mL), and the number of viable cells was measured after 3 h incubation. The bar heights show the average of 3 samples, and the error bars indicate the standard deviation. Asterisks indicate that no colony was observed.

**Fig 2:**
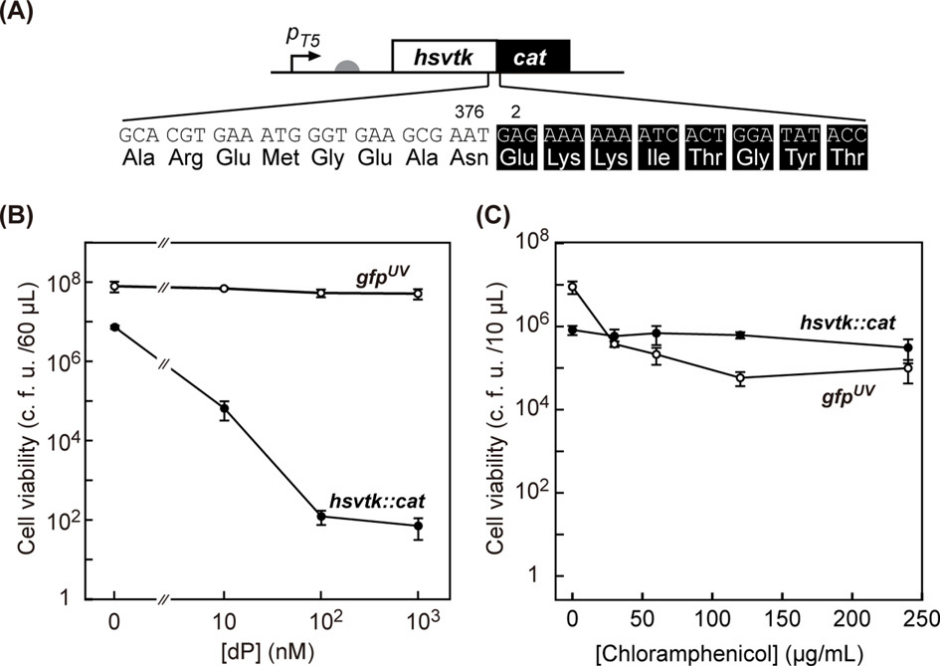
*hsvtk*::*cat* fusion gene as a ON/OFF-selector. (A) The construct for the expression of hsvTK::CAT. The reading frames of hsvTK and CAT (excluding the start codon, 217 aa) were fused without a linker, resulting in *hsvtk*::*cat*. The fusion gene was placed under the T5 promoter (*p*_*T5*_) with the translation initiation site (rbs score [25], 13843). (B) OFF-selection. *E*. *coli* MG1655 harboring either a plasmid expressing hsvTK::CAT or a plasmid expressing GFP^UV^ (negative control) were incubated with dP (0–1,000 nM), and the number of viable (colony forming) cells was measured after 3 h incubation. (C) ON-selection. *E*. *coli* MG1655 harboring either of the plasmids were treated with Cm (0–240 μg/mL), and the number of viable cells was measured after 3 h incubation. The bar heights show the average of 3 samples, and error bars indicate the standard deviation.

Approximately 10^6^ cells were obtained from an overnight culture, diluted in 0.5 mL of LB medium containing various concentrations of dP (10–1,000 nM) and shaken for 3 h at 37°C. As a reference, another batch of 10^6^ cells were shaken in the same medium without dP. The culture was rinsed with saline and plated on LB agar to recover/isolate survivor clones for further analyses. To determine the selection efficiency of the dP-selection (Figs. 1A and 2A), the number of colony-forming units in the dP-treated culture was divided by the number of colony-forming units in the non-treated culture.

### ON-selection (Km-Selection)

Approximately 10^6^ cells were obtained from an overnight culture and diluted in 0.5 mL of LB medium containing various concentrations of Km or Cm. After shaking for several hours (3–6 h) at 37°C, the culture was rinsed with saline and plated on LB agar to isolate survivors. To determine the selection efficiency, the number of colony-forming units in the culture was compared with the number of colony-forming units in the culture grown in medium without antibiotics.

### *lux* Box Library Construction

**Fig 3:**
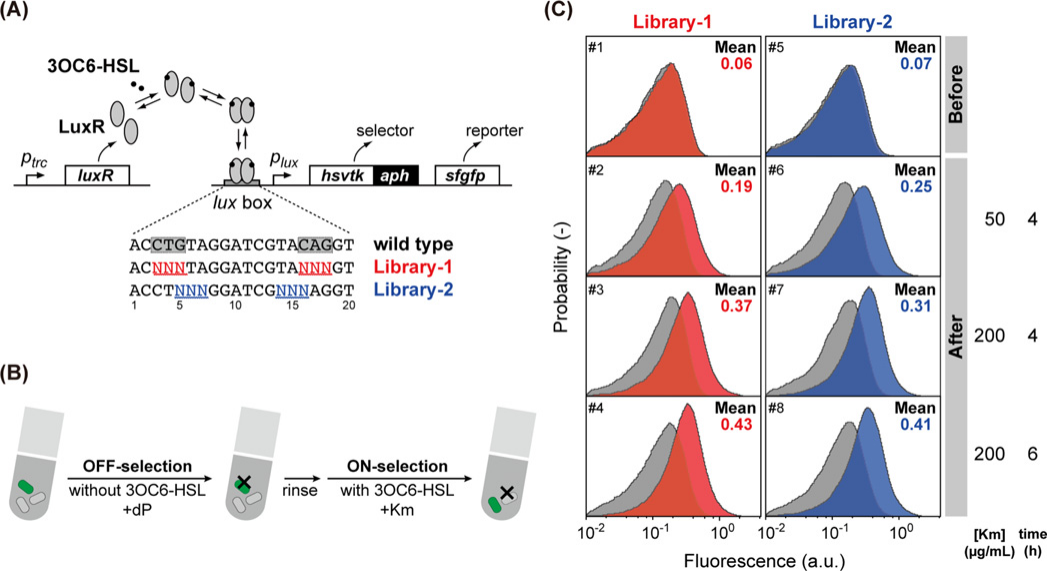
Selection of functional *lux* switches from *lux* box libraries. (A) Library design. Part (six nucleotides) of the *lux* box (LuxR-binding sites) was randomized by PCR mutagenesis, yielding Library-1 and -2. The fusion selector *hsvtk*::*aph*, together with *sfgfp*, was placed under the promoter library. LuxR was constitutively expressed from a different plasmid. (B) Selection procedure: first, approximately 10^7^ transformant cells were cultured for several hours in the presence of 1 μM dP (OFF-selection). Next, the cells were treated with 50–200 μg/mL Km (ON-selection) cultured for 1–6 hours in the presence of 1 μM 3OC6-HSL. The resultant cells were rinsed and re-grown in LB media. The cell pool was subjected to the flow cytometry in each step of this selection. (C) Flow cytometric analysis of the transformant pools before selection and after OFF/ON-selection (incubated in the absence/presence, respectively, of 1,000 nM 3OC6-HSL). Histograms in color indicate the fluorescent distribution in the presence of 3OC6-HSL, while those in gray indicate the fluorescent distribution in the absence of 3OC6-HSL.

The *lux* box libraries were constructed by oligonucleotide-based PCR mutagenesis of pAC-*plux*-*hsvtk*::*aph-sfgfp*. The randomized sites for libraries 1 and 2 are presented in Fig. 3A. Six random bases were inserted by PCR into the *lux* box of plasmid pAC-*plux-hsvtk*::*aph-sfgfp* using an ExSite-PCR-based strategy [15]. For PCR, primer P1-r (5’-TTTT GCTC TTCT CTCT AGTA TATA AACG CAGA AAGG CCCA C-3’) was used in combination with either P1-f (5’-TTTT GCTC TTCT GAGA CNNN TAGG ATCG TANN NGTT TACG CAAG AAAA TGGT TTGT TATA GTC-3’) or P2-f (5’-TTTT GCTC TTCT GAGA CCTN NNGG ATCG NNNA GGTT TACG CAAG AAAA TGGT TTGT TATA GTC-3’) (N represents an equimolar mixture of A, G, T, and C). The PCR products (7 kb) were gel-purified, self-ligated, and transformed into DH5α cells. The transformed cells were plated and grown on LB-Cm agar plates to determine the library size. Approximately 10^5^ individual colonies were obtained per transformation, which is a sufficient number to encompass all possible (4,096) variants. The cells were harvested, and plasmid DNA was recovered by mini-prep.

### Scoring the Induction Level of the *lux* Switch Using a Fluorescent Marker

A 5-μL aliquot of an overnight pre-culture was inoculated into fresh LB medium (500 μL in 96-deepwell plates with a V-shaped bottom (Greiner Bio-One, Frickenhausen, Germany)) containing various concentrations of 3OC6-HSL and the appropriate antibiotic. The mixture was then shaken for 12 h at 37°C. Cell cultures were subsequently transferred into 96-well microtiter-plates with clear bottoms (Nunc 269620, Thermo Scientific, Waltham, MA, USA). The fluorescence intensity (488 nm excitation, 507 nm emission [16]) of each sample was measured using a fluorescence microtiter-plate reader (FilterMax F5, Molecular Devices, Sunnyvale, CA, USA) with excitation at 485 nm and emission at 535 nm. The fluorescence of the cell suspension was normalized to the optical density, which was measured using the microtiter-plate reader (FilterMax F5, Molecular Devices, Sunnyvale, CA, USA) at 595 nm. The fluorescence output of the strain harboring pACmod and ptrc-*luxR* was also measured to determine the background fluorescence. All of the reported fluorescence values were obtained by subtracting this background fluorescence from the actual sample values.

## Results and Discussion

### Construction of ON/OFF Fusion Selectors

In the search for a fusion partner for *hsvtk*, we chose two positive selector genes: aminoglycoside-(3’)-phosphotransferase Type IIa (*aph*) [17] and chloramphenicol acetyltransferase type I (*cat*) [18]. Both genes have been widely used as positive selectors for plasmid maintenance and recombineering. *aph* was previously fused to *hsvtk* and used to conduct dual-selection in eukaryotic cells [12,13]. Following those reports with slight modifications, we created the fusion proteins. Specifically, we directly link the reading frame of hsvTK except the three nucleotides coding stop codon with the reading frame of positive marker APH (Fig. 1A) or CAT (Fig. 2A), excluding their start codon.

### OFF-Selection Using Fusion Selectors

The “OFF” status of genetic switches and circuits can be selected by coupling the expression of host-killing (toxic) gene elements with the device output. We recently introduced powerful negative selection systems that employ the dP-kinase activity of hsvTK [9]. The most unique feature of dP selection is the rapid nature of the selection procedure; using wild-type hsvTK, negative (OFF-state) selection can be completed in as little as 5 min and with unprecedented killing efficiency (survival rate <10^-7^). To evaluate the host-killing capability of hsvTKs fused with the positive selectors APH or CAT, *E*. *coli* MG1655 cells harboring plasmids expressing these selectors were shaken for 3 h in liquid LB medium containing varied concentrations of the nucleoside dP. Cells expressing the fusion selectors exhibited a loss of viability with increasing dP concentration, while no loss of viability was observed for the control strains (cells expressing GFP^UV^ rather than selectors) (Figs. 1B and 2B). The viability ratios of the cells expressing hsvTK::APH and hsvTK::CAT decreased to as little as 3 × 10^-7^ and 1 × 10^-5^, respectively. All of the procedures were completed under liquid handling conditions and within 3 h, demonstrating that both fusions are excellent selectors for OFF-states.

### ON-Selection Using Fusion Selectors

The “ON” status of genetic switches and circuits can be selected by coupling the expression of host-saving (*i*.*e*., detoxification or complementing growth) gene elements with the device output. To evaluate the capability of hsvTK::APH as an ‘ON-selector,’ we transformed *E*. *coli* with a plasmid encoding hsvTK::APH, and the resultant transformant was transferred into medium containing varied Km concentrations. After 3 h of incubation in the presence of 50 μg/mL Km, the cell viability count of the control strain (cells harboring a plasmid encoding GFP^UV^ rather than selectors) declined to 10^-6^ of the original value in the absence of Km (Fig. 1C). In sharp contrast, we observed no loss of viability of MG1655 cells expressing hsvTK::APH under the same conditions. Thus, hsvTK::APH can be used as a highly efficient selector for the ‘ON-state’ without overnight growth. Notably, Km is bactericidal rather than bacteriostatic [19], allowing ON-selection to be decoupled from cell growth. Positive selection using hsvTK::CAT was far less effective than positive selection using hsvTK::APH; under all tested conditions, 3 hour-treatment did not enrich hsvTK::CAT-expressing cells more than 100-fold (Fig. 2C), reflecting the bacteriostatic nature of Cm [20].

When higher (>200 μg/mL) concentrations of Km were applied to the selection medium, we observed slight decreases in the viability of cells expressing hsvTK::APH, most likely due to the titer saturation of hsvTK::APH phosphotransferase activity.

### Isolation of *lux* Promoter Variants from a Randomized Library

Having demonstrated the excellent properties of hsvTK::APH as a OFF/ON-selector, we attempted to use it to isolate a set of *lux* switches with different specifications from a combinatorial library (Fig. 3). The *lux* switch is derived from a *V*. *fischeri* quorum sensing network and has been widely used as a cell-cell communication device in synthetic biology [21–23]. In this system, the small molecule 3-oxo-hexanoyl-homoserine lactone (3OC6-HSL) was used as the cognate signaling molecule for recognition by sensor proteins (the LuxR protein family). Upon binding 3OC6-HSL, LuxR undergoes a structural transition and binds a specific DNA sequence called the *lux* box [24]. The ultimate consequence of this process is transcriptional-level switching of the genes under the control of the *lux* box by recruiting the host RNA polymerase. Single-base mutation mapping of the *lux* box has revealed that substitutions at nucleotide positions 3C, 4T, 5G, 16C, 17A, and 18G result in significant loss of activity, whereas other positions are relatively tolerant to substitution [24].

We attempted to select a functional “*lux* switch” from a library generated by partial randomization of the natural *lux* box (Fig. 3A). To explore the sequence spaces near the wild-type *lux* box and to quickly-access a series of *lux*-switches with different induction levels, two types of libraries were constructed. The six nucleotide positions, 3C, 4T, 5G, 16C, 17A, and 18G, which are most sensitive to base substitutions were randomized by PCR using primers containing N (N represents an equimolar mixture of A, G, C, and T) at the targeted positions (Library-1). Another set of nucleotide positions (5G, 6T, 7A, 14T, 15A, and 16C) was randomized in a similar manner, resulting in Library-2. It was expected that Library-2 contained higher proportion of functional *lux*-switches and with higher induction level.

The genes encoding hsvTK::APH and sfGFP were cloned in tandem downstream of the *lux* promoter library to determine their transcriptional activity (Fig. 3A). The resultant libraries (library sizes of 4,096) were transformed into cells harboring the plasmid ptrc-*luxR*. The culture was split into two, and one aliquot was maintained as a naïve library or non-selected pool, while the other was subjected to functional selection (Fig. 3B). In the absence of 3OC6-HSL, the cell culture was subjected to OFF-selection by adding dP (1,000 nM). Thereafter, the cell culture was divided into four portions, and each aliquot was subjected to ON-selection under a different condition (Km concentration and treatment time).

### Comparing Selected/Non-selected Variant Pools

Flow cytometric analyses of the initial pools (naïve libraries) demonstrated that most of the variants were non-functional (Fig. 3C, panels 1 and 5); the fluorescence of the population was virtually unchanged upon induction (addition of 3OC-6HSL), indicating that the majority of the *lux* box libraries were not recognized by LuxR. We observed a small number of transformants that were always fluorescent (even without induction) in the OFF-selected pool (data not shown). Such clones were likely attributable to those with inactivating mutations in *hsvtk* gene accumulated during PCR. These variants were eliminated from the pool after ON-selection, indicating that mutations inactivating the OFF-selector sequence (*hsvtk*) likely accompanied the inactivation of the ON-selector gene (*aph*), and thus these variants could not survive subsequent ON-selection. After ON-selection, the library consisted exclusively of 3OC6-HSL-induced switching variants (Fig. 3C, panels 2–4 and 6–8 for Library-1 and Library-2, respectively). Notably, the average fluorescence score was higher under inducing conditions, consistent with the selection pressure (Km concentration and treatment time), indicating that the selection threshold in the induction level can be adjusted using the selection condition.

From each resultant library after OFF-/ON-selection, we evaluated the expression level of 45 randomly selected clones from each selected population by measuring GFP fluorescence using a plate reader. We observed a significant distribution in the expression level under inducing conditions (adding 3OC6-HSL) in each selected pool, while the cut-off induction level was higher for those ON-selected under more stringent conditions (S1 Fig.). Most of the selected variants exhibited low levels of non-induced (leaky) expression; the dP-kinase activity of hsvTK is very potent, enabling strong counter-selection against leaky switches [9] and ensuring the high stringency of the isolated mutants.

### Isolation of a Series of *lux* Promoters with Different Strengths

**Fig 4:**
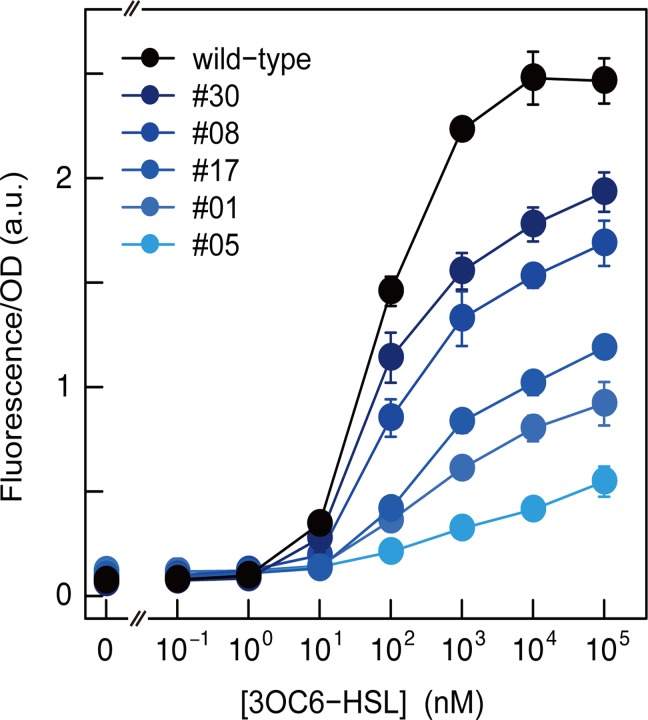
Dose-response of *lux* promoter variants to 3OC6-HSL activated by LuxR. Five individual *lux* promoter variants (#30, #08, #17, #01, and #05) were selected from 45-randomly picked ones from the pool shown in S1 Fig. (C) (variants recovered from OFF-selected Library-2 that survived the ON selection (incubation with 50 μg/mL of Km for 4 h)) and quantitatively characterized using micro-titer plate measurement of sfGFP expression after 12 h growth in medium containing various concentrations of 3OC6-HSL (0–10^5^ nM). The bar heights show the average of 3 samples, and the error bars indicate the standard deviation.

Among the selected pools, the pool subjected to the least stringent ON-selection (50 μg/mL Km for 4 h) treatment (panel 6 in Fig. 3C and highlighted variants in S1 Fig.) exhibited the greatest phenotypic diversity. More mutable positions were randomized to generate Library-2 compared to Library-1, and this pool comprised the survivors of the least stringent selection conditions conducted in this experiment. From this pool, we isolated five variants with different induction levels (promoter strength) and analyzed their transfer function to 3OC6-HSL (Fig. 4; the sequences are provided in S2 Table). All five variants exhibited 3OC6-HSL-induced increases in expression, and their induction levels (promoter strength) differed (ranging from 4.5- to 32-fold induction). Thus, we rapidly obtained a series of variants with different promoter strengths. Because the switch variants were generated by mutating the promoter (*lux* box) sequence, other specifications, such as ligand sensitivity and stringency/leakiness, under non-induced conditions were similar.

This study demonstrated that hsvTK::APH enables the rapid and tunable selection of desirable genetic switches. All procedures were completed using liquid handling, suggesting that different selection experiments can be conducted in parallel in a multi-well format. Because the selection mechanisms are not based on growth rate differences, ON-state selection and OFF-state selection does not require overnight growth. Further optimization of this protocol would greatly facilitate the evolutionary design of complex genetic circuits, in which multiple selections must be repeated for various input/output states.

## Supporting Information

S1 Fig Characterization of *lux* switches randomly picked from the selected pool with different selection conditions. Individual variants were picked from (**a**) Library-1 (Km 50 μg/mL, 4 h), (**b**) Library-1 (Km 200 μg/mL, 4 h), (**c**) Library-2 (Km 50 μg/mL, 4 h), and (**d**) Library-2 (Km 200 μg/mL, 4 h) and quantitatively characterized using microtiter plate measurement of sfGFP expression after 12 h growth in medium in the presence (open bar) or absence (solid bar) of 3OC6-HSL (1,000 nM). The variants highlighted were analyzed for the dose-dependency on 3OC6-HSL (Fig. 4), and the moieties including *lux* box sequences were PCR-amplified for the sequence analysis.
(TIF)

S1 Table Plasmids used in this study. (PDF)

S2 Table Sequence analysis of *lux* box sequences of the five selected variants (Panel 6 in Fig. 3C). Five variants isolated from the survivor pool that went through dP-selection in the absence of 3OC6-HSL and then the ON-selection in the presence of 3OC6-HSL with 50 μg/mL of Km for 4 hours (Panel 6 in Fig. 3C).
(PDF)

## References

[1] Haseltine EL, Arnold FH. Synthetic gene circuits: design with directed evolution. Annu Rev Biophys Biomol Struct. 2007;36: 1–19. 17243895 10.1146/annurev.biophys.36.040306.132600

[2] Schaerli Y, Isalan M. Building synthetic gene circuits from combinatorial libraries: screening and selection strategies. Mol Biosyst. 2013;9: 1559–1567. 10.1039/c2mb25483b 23340599

[3] Yokobayashi Y, Arnold FH. A dual selecion module for directed evolution of genetic circuits. Natural Computing. 2005;4: 245–254. 16192677

[4] Collins CH, Leadbetter JR, Arnold FH. Dual selection enhances the signaling specificity of a variant of the quorum-sensing transcriptional activator LuxR. Nat Biotechnol. 2006;24: 708–712. 16715074 10.1038/nbt1209

[5] Muranaka N, Sharma V, Nomura Y, Yokobayashi Y. An efficient platform for genetic selection and screening of gene switches in *Escherichia coli* . Nucleic Acids Res. 2009;37: e39 10.1093/nar/gkp039 19190095 PMC2655682

[6] Nomura Y, Yokobayashi Y. Dual selection of a genetic switch by a single selection marker. Biosystems. 2007;90: 115–120. 16942834 10.1016/j.biosystems.2006.07.006

[7] Topp S, Gallivan JP. Random walks to synthetic riboswitches: a high-throughput selection based on cell motility. ChemBioChem. 2008;9: 210–213. 18098254 10.1002/cbic.200700546

[8] Rackham O, Chin JW. A network of orthogonal ribosome-mRNA pairs. Nat Chem Biol. 2005;1: 159–166. 16408021 10.1038/nchembio719

[9] Tashiro Y, Fukutomi H, Terakubo K, Saito K, Umeno D. A nucleoside kinase as a dual selector for genetic switches and circuits. Nucleic Acids Res. 2011;39: e12 10.1093/nar/gkq1070 21062820 PMC3035434

[10] Patterson AV, Saunders MP, Greco O. Prodrugs in genetic chemoradiotherapy. Curr Pharm Des. 2003;9: 2131–2154. 14529410 10.2174/1381612033454117

[11] Negishi K, Loakes D, Schaaper RM. Saturation of DNA mismatch repair and error catastrophe by a base analogue in *Escherichia coli* . Genetics. 2002;161: 1363–1371. 12196386 10.1093/genetics/161.4.1363 PMC1462219

[12] Candotti F, Agbaria R, Mullen CA, Touraine R, Balzarini J, Johns DG, et al Use of a herpes thymidine kinase/neomycin phosphotransferase chimeric gene for metabolic suicide gene transfer. Cancer Gene Ther. 2000;7: 574–580. 10811475 10.1038/sj.cgt.7700153

[13] Schwartz F, Maeda N, Smithies O, Hickey R, Edelmann W, Skoultchi A, et al A dominant positive and negative selectable gene for use in mammalian cells. Proc Natl Acad Sci U S A. 1991;88: 10416–10420. 1720540 10.1073/pnas.88.23.10416 PMC52939

[14] Schmidt-Dannert C, Umeno D, Arnold FH. Molecular breeding of carotenoid biosynthetic pathways. Nat Biotechnol. 2000;18: 750–753. 10888843 10.1038/77319

[15] Hemsley A, Arnheim N, Toney MD, Cortopassi G, Galas DJ. A simple method for site-directed mutagenesis using the polymerase chain reaction. Nucleic Acids Res. 1989;17: 6545–6551. 2674899 10.1093/nar/17.16.6545 PMC318348

[16] Pedelacq JD, Cabantous S, Tran T, Terwilliger TC, Waldo GS. Engineering and characterization of a superfolder green fluorescent protein. Nat Biotechnol. 2006;24: 79–88. 16369541 10.1038/nbt1172

[17] Davies J, Smith DI. Plasmid-determined resistance to antimicrobial agents. Annual Reviews in Microbiology. 1978;32: 469–508. 10.1146/annurev.mi.32.100178.002345 360974

[18] Shaw WV. Chloramphenicol acetyltransferase: enzymology and molecular biology. CRC Crit Rev Biochem. 1983;14: 1–46. 6340955 10.3109/10409238309102789

[19] Garrett ER, Won CM. Kinetics and mechanisms of drug action on microorganisms. XVII. Bactericidal effects of penicillin, kanamycin, and rifampin with and without organism pretreatment with bacteriostatic chloramphenicol, tetracycline, and novobiocin. J Pharm Sci. 1973;62: 1666–1673. 4584653 10.1002/jps.2600621018

[20] Brock TD. Chloramphenicol. Bacteriol Rev. 1961;25: 32–48. 16350168 10.1128/br.25.1.32-48.1961 PMC441072

[21] Basu S, Mehreja R, Thiberge S, Chen MT, Weiss R. Spatiotemporal control of gene expression with pulse-generating networks. Proc Natl Acad Sci U S A. 2004;101: 6355–6360. 15096621 10.1073/pnas.0307571101 PMC404049

[22] Brenner K, Karig DK, Weiss R, Arnold FH. Engineered bidirectional communication mediates a consensus in a microbial biofilm consortium. Proc Natl Acad Sci U S A. 2007;104: 17300–17304. 17959781 10.1073/pnas.0704256104 PMC2077251

[23] Tamsir A, Tabor JJ, Voigt CA. Robust multicellular computing using genetically encoded NOR gates and chemical 'wires'. Nature. 2011;469: 212–215. 10.1038/nature09565 21150903 PMC3904220

[24] Antunes LC, Ferreira RB, Lostroh CP, Greenberg EP. A mutational analysis defines *Vibrio fischeri* LuxR binding sites. J Bacteriol. 2008;190: 4392–4397. 18083819 10.1128/JB.01443-07 PMC2446796

[25] Salis HM, Mirsky EA, Voigt CA. Automated design of synthetic ribosome binding sites to control protein expression. Nat Biotechnol. 2009;27: 946–950. 10.1038/nbt.1568 19801975 PMC2782888

