# Supplementary figures and images for: Rapid and Liquid-Based Selection of Genetic Switches Using Nucleoside Kinase Fused with Aminoglycoside Phosphotransferase
Source: PLoS One. 2015 Mar 19;10(3):e0120243. doi: 10.1371/journal.pone.0120243 (PMC4366196; doi:10.1371/journal.pone.0120243)

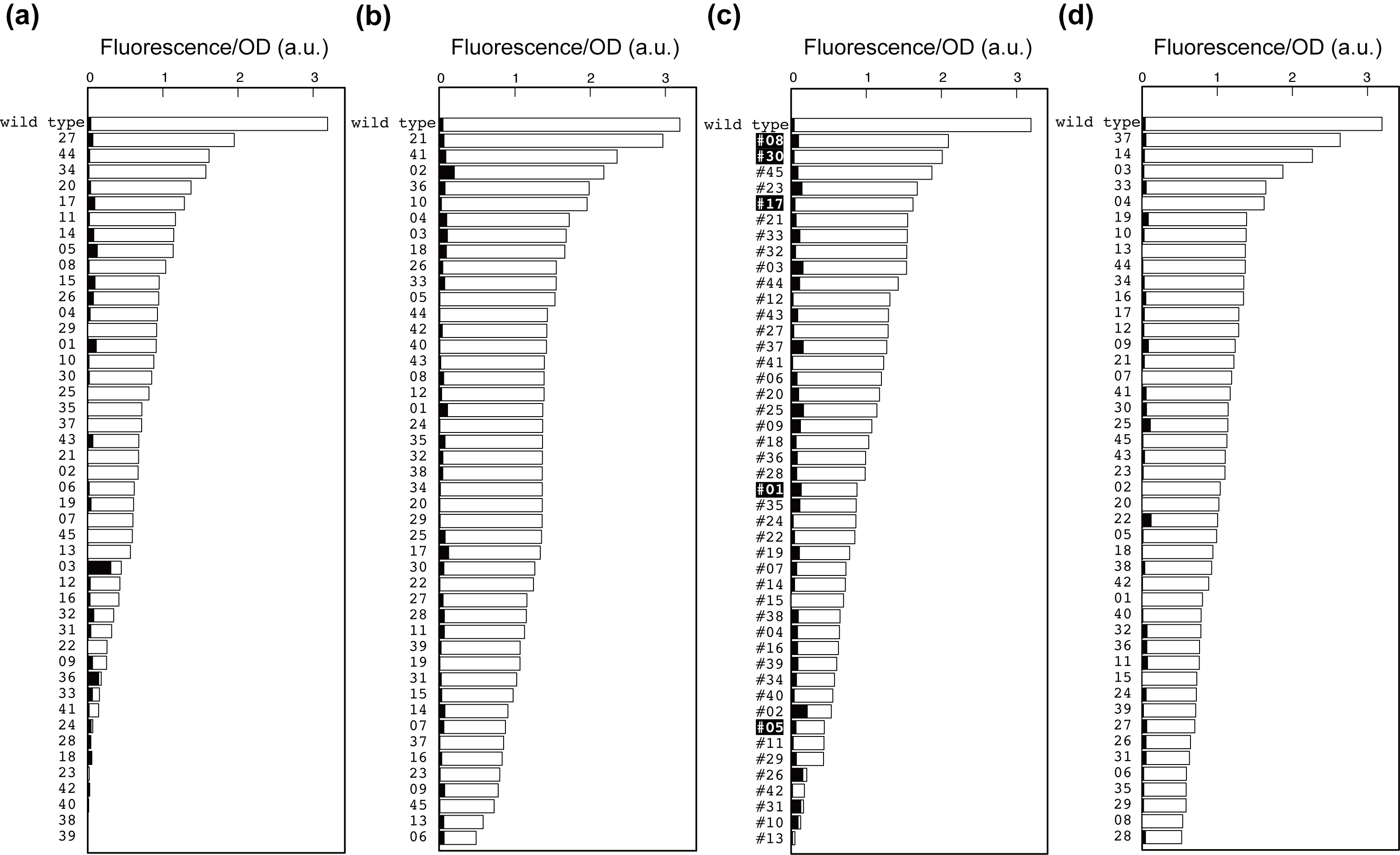

Supplement: S1 Fig — Individual variants were picked from (a) Library-1 (Km 50 μg/mL, 4 h), (b) Library-1 (Km 200 μg/mL, 4 h), (c) Library-2 (Km 50 μg/mL, 4 h), and (d) Library-2 (Km 200 μg/mL, 4 h) and quantitatively characterized using microtiter plate measurement of sfGFP expression after 12 h growth in medium in the presence (open bar) or absence (solid bar) of 3OC6-HSL (1,000 nM). The variants highlighted were analyzed for the dose-dependency on 3OC6-HSL (Fig. 4), and the moieties including lux box sequences were PCR-amplified for the sequence analysis. (TIF) [file pone.0120243.s001.tif]
