# Supplementary material for: Rapid and Liquid-Based Selection of Genetic Switches Using Nucleoside Kinase Fused with Aminoglycoside Phosphotransferase
Source: PLoS One. 2015 Mar 19;10(3):e0120243. doi: 10.1371/journal.pone.0120243 (PMC4366196; doi:10.1371/journal.pone.0120243)
Supplement: S1 Table — (PDF) [file pone.0120243.s002.pdf]

Table S1. Plasmids used in this study.

Plasmid name	vector	Ori/marker	Source
pJ204-*pT5- hsvTK::aph*	pJ204	*ColE1*/*bla*	This study
pJ204-*pT5- hsvTK::cat*	pJ204	*ColE1*/*bla*	This study
pJ204-*pT5- gfp*^{UV}	pJ204	*ColE1*/*bla*	This study
pTrc-*luxR*	pTrcHis2	*ColE1*/*bla*	This study
pAC-*plux-hsvTK*::*aph-sfgfp*	pACmod	*p15A*/*cat*	This study
pACmod	pACmod	*p15A*/*cat*	[14]
