# Supplementary material for: Rapid and Liquid-Based Selection of Genetic Switches Using Nucleoside Kinase Fused with Aminoglycoside Phosphotransferase
Source: PLoS One. 2015 Mar 19;10(3):e0120243. doi: 10.1371/journal.pone.0120243 (PMC4366196; doi:10.1371/journal.pone.0120243)
Supplement: S2 Table — Five variants isolated from the survivor pool that went through dP-selection in the absence of 3OC6-HSL and then the ON-selection in the presence of 3OC6-HSL with 50 μg/mL of Km for 4 hours (Panel 6 in Fig. 3C). (PDF) [file pone.0120243.s003.pdf]

Table S2. Sequence analysis of *lux* box sequences of the five selected variants (Panel 6 in Figure 3C).

Five variants isolated from the survivor pool that went through dP-selection in the absence of 3OC6-HSL and then the positive selection in the presence of 3OC6-HSL with 50 µg/mL of Km for 4 hours (Panel 6 in Figure 3C).

variant	*lux* box sequence
WT	ACCTGTAGGATCGTACAGGT
#01	ACCTGT**T**GGATCG**GT**CAGGT
#05	ACCTGT**T**GGATCGT**C**CAGGT
#08	ACCTGT**T**GGATCGTACAGGT
#17	ACCTGT**C**GGATCGT**T**CAGGT
#30	ACCTGTAGGATCGT**G**CAGGT
